# Citrullinated Histone H3 as a Therapeutic Target for Endotoxic Shock in Mice

**DOI:** 10.3389/fimmu.2019.02957

**Published:** 2020-01-09

**Authors:** Qiufang Deng, Baihong Pan, Hasan B. Alam, Yingjian Liang, Zhenyu Wu, Baoling Liu, Nirit Mor-Vaknin, Xiuzhen Duan, Aaron M. Williams, Yuzi Tian, Justin Zhang, Yongqing Li

**Affiliations:** ^1^Xiangya Hospital, Central South University, Changsha, China; ^2^Department of Surgery, University of Michigan, Ann Arbor, MI, United States; ^3^The First Hospital, China Medical University, Shenyang, China; ^4^Division of Infectious Diseases, Department of Internal Medicine, University of Michigan, Ann Arbor, MI, United States; ^5^Department of Pathology, Loyola University Medical Center, Maywood, IL, United States

**Keywords:** citrullinated histone H3, endotoxic shock, neutrophil extracellular traps, new anti-CitH3 antibody, inflammation, acute lung injury, survival

## Abstract

Sepsis results in millions of deaths every year, with acute lung injury (ALI) being one of the leading causes of mortality in septic patients. As neutrophil extracellular traps (NETs) are abundant in sepsis, neutralizing components of NETs may be a useful strategy to improve outcomes of sepsis. Citrullinated histone H3 (CitH3) has been recently shown to be involved in the NET formation. In this study, we demonstrate that CitH3 damages human umbilical vein endothelial cells (HUVECs) and potentiates NET formation through a positive feedback mechanism. We developed a novel CitH3 monoclonal antibody to target peptidylarginine deiminase (PAD) 2 and PAD 4 generated CitH3. In a mouse model of lethal lipopolysaccharide (LPS) induced shock, neutralizing CitH3 with the newly developed anti-CitH3 monoclonal antibody attenuates inflammatory responses, ameliorates ALI, and improves survival. Our study suggests that effectively blocking circulating CitH3 might be a potential therapeutic method for the treatment of endotoxemia.

## Introduction

Sepsis is defined as a life-threatening organ dysfunction caused by the dysregulation of host response secondary to infection (1). Acute lung injury (ALI) develops in nearly 40% of the septic patients and is one of the leading causes of death (2).

Excessive neutrophil extracellular traps (NETs) have been detected in sepsis and are associated with significant organ injury (3). NETs are stranded, decondensed DNA (deoxyribonucleic acid) fibers accompanied by intracellular proteins, including histones, neutrophil elastase (NE), myeloperoxidase (MPO), and other proteins coming from various neutrophil organelles (4, 5). Histone citrullination/deimination induced by peptidyl arginine deiminases (PADs) is an important posttranslational modification that facilitates chromatin decondensation during NET formation (6). Moreover, citrullinated histones are found in the extracellular space of neutrophils along with DNA as components of NETs (7).

NETs are generally regarded as a double-edged sword (8). NETs can immobilize and kill a broad range of pathogens: gram-positive and -negative bacteria, fungi, viruses, and protozoa (4, 9–14). However, they also promote tissue damage, increase thrombosis, and cause disruption of the autoimmune system (15). Strategies targeting NET formation or NET components have proven therapeutic in animal models of sepsis. PAD inhibitors, which can disrupt NET formation, have been shown to protect animals from endotoxic shock or septic shock. Administration of a pan-PAD Inhibitor, Cl-amidine, improves survival in both lethal and sub-lethal models of murine sepsis, increases bacterial clearance, and ameliorates thymus and bone marrow atrophy (16, 17). YW3-56, another pan-PAD inhibitor, has been shown to increase survival in mice with lipopolysaccharide (LPS)-induced endotoxic shock (18). Administration of DNase has been demonstrated to markedly reduce cell-free DNA and improve outcomes in both *Escherichia coli*-induced and CLP-induced sepsis (19, 20). Neutralization of histone H4, a NET component, has been shown to significantly reduce the mortality in mouse models of cecal ligation and puncture (CLP) (21).

Citrullinated histone H3 (CitH3) has been shown to be highly involved in the process of NETosis (4, 6, 18, 22). As such, CitH3 is considered a good biomarker for the diagnosis of endotoxic shock due to its early appearance (as early as 30 min) in the blood, long half-life, high specificity, and response to treatment (23). However, the physio-pathologic role of CitH3 in sepsis has not been well-defined. Furthermore, it is unknown whether CitH3 could be considered a therapeutic target. In this study, we investigated the adverse effects of CitH3 and developed a new anti- histone H3 (citrullinated R2+R8+R17+R26) monoclonal antibody [CitH3 mAb (4 Cit)] utilizing CitH3 with four citrullines (4 Cit) as the antigen. We then evaluated this novel antibody in a murine model of endotoxic shock to explore the therapeutic value of neutralizing the circulating CitH3 protein.

## Materials and Methods

### Generation of CitH3 mAb (4 Cit)

CitH3 peptide with four citrulline residues [A(Cit)TKQTA(Cit) KSTGGKAP(Cit) KQLATKAA(Cit)KSAP], referred to as CitH3 (R2+R8+R17+R26) peptide, was chemically synthesized by New England Peptide, Inc. (Gardner, MA, USA) and utilized to generate the 4 Cit monoclonal antibody in ProMab Biotechnologies, Inc. (Richmond, CA, USA) using the company-approved animal protocol. Balb/c mice were immunized with the CitH3 (R2+R8+R17+R26) peptide. Antibody titers were then determined by enzyme-linked immunosorbent assay (ELISA). Splenocytes from the mouse with the highest antibody titer were fused with myeloma cells (SP2/0) to generate hybridomas. Anti-CitH3 (R2+R8+R17+R26) peptide-specific hybridomas were identified using ELISA against the CitH3 (R2+R8+R17+R26) peptide. One to two million viable hybridoma cells were injected into a mouse peritoneal cavity to produce ascites, which was harvested, and particles were removed through centrifugation. Protein G purification was performed to get the final CitH3 mAb (4 Cit).

### Cell Culture and Treatment

Human umbilical vein endothelial cells (HUVECs) (Lonza, Walkersville, MD, USA) were cultured using endothelial cell growth medium (EGM) BulletKit (Lonza, Walkersville, MD, USA). HUVECs (5 × 10^5^ cells/ml) were grown on 12-mm Transwells with 0.4 μm pore polyester membrane inserts (Corning Life Sciences, Corning, NY, USA) for 3 days to develop a confluent (90%) monolayer. HUVECs were then treated for 16 h with 5 μg/ml histone H3 peptide (ARTKQTARKSTGGKAPRKQLATKAARKSAP) or CitH3 peptide [A(Cit)TKQTA(Cit) KSTGGKAP(Cit)KQLATKAA(Cit)KSAP]. Chambers were then incubated in the presence of 1 mg/ml 10-kDa FITC-dextran (Thermo Scientific, Rockford, IL, USA). The fluorescence of media in the lower chambers was measured by a GloMax-multi detection system (Promega, Madison, WI, USA).

### Mice

Male C57BL/6 mice (7–8 weeks) were purchased from The Jackson Laboratory (Bar Harbor, ME, USA) and housed for at least 3 days with food and water *ad libitum* before the experiment. All experiments were performed in compliance with the animal welfare and research regulations. The animal protocol for this study was approved by the University of Michigan Institutional Animal Care and Use Committee.

### Lethal Endotoxic Shock and Antibody Treatment

LPS was injected intraperitoneally (20 mg/kg), inducing lethal endotoxic shock in the mice. Either CitH3 mAb (4 Cit) (about 20 mg/kg) or the same amount of anti-histone H3 [(citrullinated R2+R8+R17) monoclonal antibody (CitH3 mAb (3 Cit), Item number 9003062 with Batch numbers 0515031-1, 0513766-1, and 0516044-1; Cayman Chemical, Ann Arbor, MI, USA)] was administered via tail vein injection. Mouse receiving immunoglobulin G (IgG) only (20 mg/kg) or LPS followed by IgG served as controls (*n* = 9/group). Survival was monitored for 10 days. Kaplan–Meier curves were used to compare the survival rates.

In another cohort, mice were also randomly divided into four groups: (1) IgG only (20 mg/kg), (2) LPS (20 mg/kg) + IgG (20 mg/kg), (3) LPS + CitH3 mAb (4 Cit) (20 mg/kg), and (4) LPS + CitH3 mAb (3 Cit) (20 mg/kg) (*n* = 3/group). Animals were sacrificed 12 h after treatment (*n* = 3), and organs were collected and stored in −80°C for further use. Blood samples were at room temperature (RT) for 1 h to allow for clotting and separation of serum. Serum was collected by centrifugation of the clotted blood at 3,000 × *g* at 4°C for 20 min, and then stored immediately at −80°C.

### Western Blotting for Antibody Validation

One-half microgram of five different peptides [H3, AceH3, CitH3 (R2+R8+R17+R26), CitH3 (R26), and MetH3] or 3 ng of CitH3 protein was subjected to SDS-polyacrylamide gel electrophoresis and was transferred onto a nitrocellulose membrane. Membranes were then probed with the same concentration (2 μg/ml) of CitH3 mAb (4 Cit) or CitH3 mAb (3 Cit). Donkey anti-mouse 800 CW antibodies (LI-COR, Lincoln, NE, USA) were used as the secondary detection antibodies (1: 5,000 dilution). Finally, the membranes were exposed to 800 channel Odyssey Imaging System (LI-COR, Lincoln, NE, USA). Immunoblot signal intensity was analyzed using Image Studio Lite (LI-COR, Lincoln, NE, USA).

### CitH3 ELISA

A “sandwich” ELISA, which has been developed by our laboratory and described previously (23), was used. In brief, 0.5 μg/well CitH3 mAb (4 Cit) or CitH3 mAb (3 Cit) was coated in 96-well plates (Corning Life Sciences, Corning, NY, USA) at 4°C overnight and then blocked with 100 μl of protein-free blocking buffer (Thermo Scientific, Rockford, IL, USA) at 4°C overnight. The wells were then incubated with CitH3 (R2+R8+R17+R26) peptide or mouse serum (1:1 diluted in blocking buffer) at RT for 2 h, followed by rabbit anti-CitH3 polyclonal antibody (1:3,000 diluted, Abcam, Cambridge, MA, USA) incubation for 2 h at RT. Next, 96-well plates were probed with donkey anti-rabbit horseradish peroxidase (HRP) conjugate IgG (1:50,000 diluted, Jackson ImmunoResearch, West Grove, PA, USA). 3,3′,5,5′-Tetramethylbenzidine (TMB, Thermo Fisher Scientific, Waltham, MA, USA) was utilized to develop the plate for 30 min at RT in the dark before adding stop solution (R&D Systems Inc., Minneapolis, MN, USA). Absorbance was measured at 450 nm.

### Cytokines

Levels of pro-inflammatory cytokines in the serum or lung homogenates were measured by ELISA. IL-1β was measured using the Mouse IL-1β/IL-1F2 DuoSet ELISA (R&D Systems, Minneapolis, MN, USA) and TNF-α was detected using Mouse TNF-α DuoSet ELISA (R&D Systems, Minneapolis, MN, USA). The ELISA was performed blindly by an independent researcher.

### Histopathology

Twelve hours after treatment, lung samples were collected and fixed with 4% paraformaldehyde, and then dehydrated in 70% ethanol. The lung tissues were embedded in paraffin and cut into 5-μm sections. Hematoxylin–eosin staining was performed by a blinded researcher. Histological analysis of ALI was also graded by a blinded pathologist with a scale from 0 to 3 among the following domains: (1) septal mononuclear cell/lymphocyte infiltration, (2) septal hemorrhage and congestion, (3) neutrophils, (4) alveolar macrophages, (5) alveolar hemorrhage, and (6) alveolar edema (0: “absent,” 1: “mild,” 2: “moderate,” and 3: “severe”). The total injury score was calculated by adding up the scores for all parameters.

### Human Neutrophil Isolation and Treatment

Collection of blood samples from a healthy human volunteer was approved by the Institutional Review Board (IRB) of the University of Michigan (HUM00048623). Neutrophil isolation has been described previously (24). Briefly, human whole blood up to 60 ml was placed in 7 ml 0.25 M citrate solution and 10 ml 6% dextran solution in PBS. After incubation for 30 min, the upper phase was collected and layered on 15 ml of Histopaque-1077 (Sigma, St. Louis, MO, USA), and separated through centrifugation for 30 min at 800 × *g* at RT. The pellet was resuspended with 3 ml of cold PBS and layered on 12 ml of Histopaque-1119 (Sigma, St. Louis, MO, USA) and centrifuged again for 30 min at 800 × *g* at RT. The neutrophil layer was then transferred to a 50-ml tube and 40 ml of PBS was added. After 10 min centrifugation at 500 × *g*, 4°C, the supernatant was disposed and the neutrophil fraction was suspended in RPMI 1640 supplemented with 2% BSA to make the final neutrophil concentration at 500,000 cells/ml. One milliliter cell solution was added to each 22 × 22, 1.5/2.5 glass coverslip that has been treated with 0.001% poly-L-lysine (Sigma, St. Louis, MO, USA). After cell adherence, neutrophils were treated for 2 h with 5 μg/ml CitH3 peptide [A(Cit)TKQTA(Cit) KSTGGKAP(Cit)KQLATKAA(Cit)KSAP] or H3 peptide (ARTKQTARKSTGGKAPRKQLATKAARKSAP) as control. Media was removed before immunocytochemistry.

### Immunocytochemistry

Cells were washed before fixation in 3.7% paraformaldehyde/PBS for 10 min at RT. After three washes with PBS for 5 min each, cells were blocked in 2% BSA/PBS overnight at 4°C. Neutrophils were then probed overnight at 4°C with anti-CitH3 monoclonal antibody (Cayman Chemical, Ann Arbor, Michigan, USA) at a dilution of 1: 1,000 and anti-MPO polyclonal antibody (Abcam, Cambridge, MA, USA) at the dilution of 1:100 in 10% normal donkey serum. Cells were washed with PBS three times for 5 min each. Next, cells were incubated at RT for 1 h with FITC-conjugated donkey anti-rabbit and TRITC-conjugated donkey anti-mouse IgG (Jackson ImmunoResearch Laboratories, West Grove, PA, USA) at a dilution of 1:300 in 10% normal donkey serum. Following another three washes with PBS, the coverslips were mounted with anti-fade reagent with DAPI (Thermo Fisher Scientific, Waltham, MA, USA). Eight fields each group were selected randomly for further quantification of NETs formation. Fluorescence microscopy images were analyzed with Image J software to count the number of NETs induced by peptides per 100 neutrophils.

### Measurement of Serum Levels of dsDNA

The PicoGreen assay kit (Invitrogen, San Diego, CA, USA) was used to detect circulating dsDNA per manufacturer's instruction.

### Statistical Analysis

Analyses were performed with GraphPad Prism 7 (GraphPad Software Inc., La Jolla, CA, USA). Data are presented as mean ± standard error of mean (SEM). Log-rank test was used to analyze the survival curve. One-way analysis of variance (ANOVA) followed by Bonferroni's multiple comparison test was used for comparisons between three or more groups. Mann–Whitney *U* test (non-parametric test) was performed for comparisons between two groups. A *p* < 0.05 was considered statistically significant. ^*^p < 0.05; **p < 0.01; ***p < 0.001; ****p < 0.0001.

## Results

### CitH3 Increases HUVEC Permeability and Induces NET Formation

CitH3 is undetectable in the serum and peritoneal fluid under normal physiologic conditions in mice; however, it is elevated in the samples obtained from endotoxic or septic mice (17, 23). The function of CitH3 remains largely unknown. It was recently reported that human recombinant CitH3 protein could disrupt endothelial barrier *in vivo*, probably through opening cell–cell adheres junctions and reorganizing the actin cytoskeleton (25). This conclusion needs further evaluation since the recombinant CitH3 protein was catalyzed by PAD4 purified from *E. coli*, and endotoxin contamination can compromise the validity of the experimental results (26, 27).

**Figure 1:**
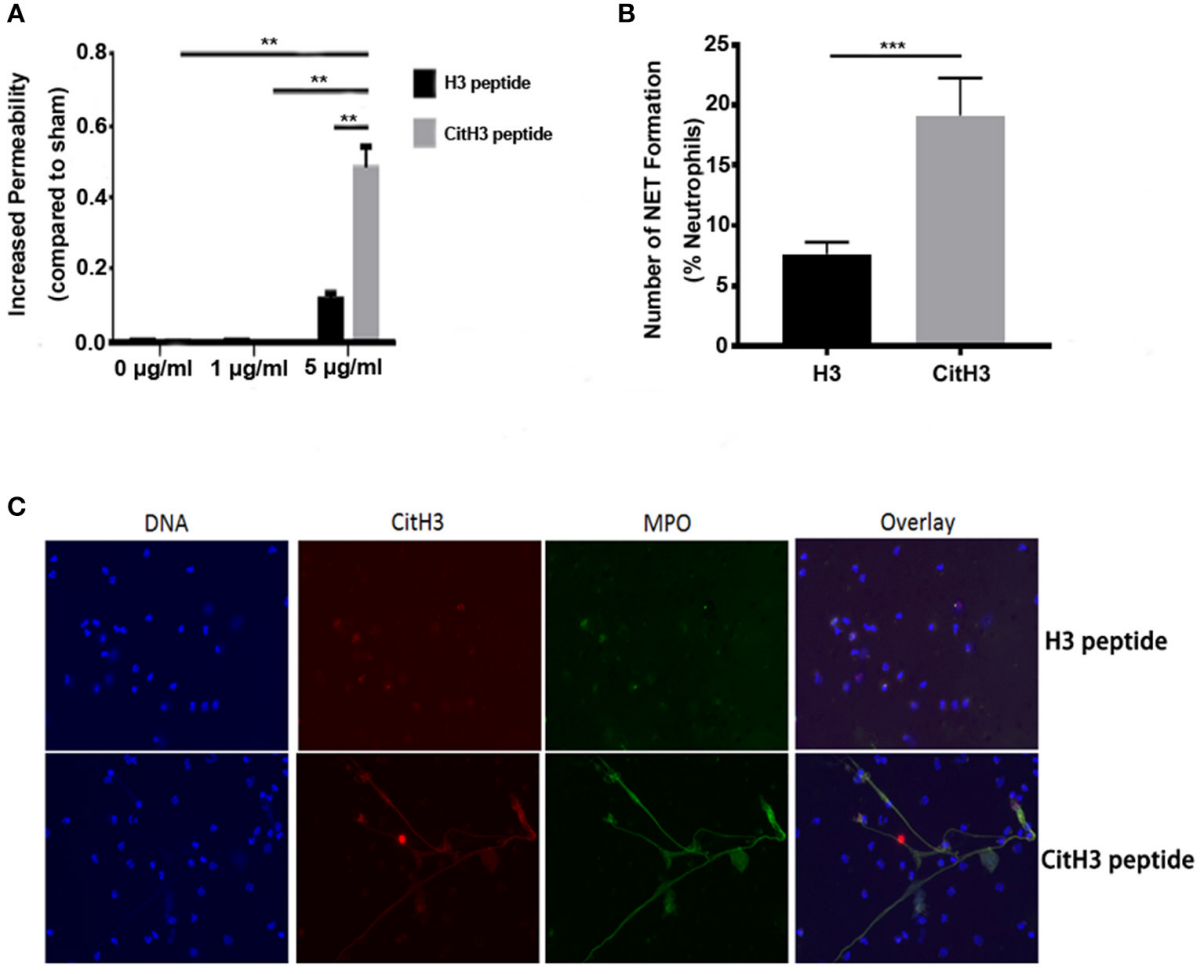
CitH3 induces vascular leakage and formation of NETs. **(A)** HUVECs were treated with 1 μg/ml or 5 μg/ml of H3 peptide or CitH3 peptide for 16 h after forming a confluent (90%) monolayer on Transwells. Chambers were then incubated in 1 mg/ml 10-kDa FITC-dextran. The fluorescence of media in the lower chambers were presented as means ± SEM (*n* = 5/group). **(B,C)** Human neutrophils were purified from the peripheral blood of a healthy volunteer. After 2 h treatment with 5 μg/ml CitH3 peptide, or H3 peptide as a control (*n* = 3/group), neutrophils were stained with DAPI (blue), mouse anti-CitH3 (red), and rabbit anti-MPO (green) antibodies. Neutrophils stimulated with CitH3 peptide formed NETs, whereas those treated with H3 peptide did not. Fluorescence microscopy images were analyzed with Image J software to count the number of NETs induced by peptides per 100 neutrophils. **p < 0.01; ***p < 0.001.

In the present study, synthesized CitH3 peptide was used. It was found that the synthesized CitH3 peptide (5 μg/ml) significantly increased (*p* < 0.01) dextran leakage from HUVECs compared to sham control or 1 μg/ml CitH3 treatment (Figure 1A). HUVECs treated with 5 μg/ml H3 peptide had increased dextran leakage compared to sham; however, it was significantly lower (*p* < 0.01) compared to CitH3 at the same concentration (5 μg/ml). The result suggests that CitH3 might be more toxic to HUVECs than H3, implicating the adverse effect of CitH3 in sepsis. Since CitH3 could induce prominent leakage from endothelial cells, neutralizing CitH3 may be beneficial in endotoxic shock or sepsis.

Human neutrophils incubated with CitH3 peptide also produced NETs after a short incubation period of 2 h. To visualize NETs, cells were co-stained with MPO, CitH3, and DNA. As shown in Figure 1C, there were no NETs detected in neutrophils treated with H3 peptide; however, NET structures were observed following CitH3 treatment, including stretched DNA filaments along with MPO and CitH3. As such, CitH3 increases HUVEC permeability and can induce NET formation *ex vivo*.

### The CitH3 mAb (4 Cit) Recognizes Histone H3 Citrullinated R26 and Has Higher Binding Capacity Compared to the Antibody Against Histone H3 [Citrullinated R2+R8+R17 (CitH3 mAb (3 Cit))]

Although there are some commercially available antibodies that bind to citrulline residues in histones, their efficacy has been found to be inconsistent (28), and there is a need for more reliable anti-CitH3 antibodies. In addition, the commercially available anti-CitH3 mAb only recognizes histone H3 citrullinated R2+R8+R17 [CitH3 mAb (3 Cit)], for which PAD4 is responsible (29). However, histone H3 R26 can also be citrullinated, by PAD2 but not PAD4 (30). Therefore, utilizing the CitH3 peptide with four citrulline residues at histone 2+8+17+26, we developed the new mouse anti-CitH3 monoclonal antibody, referred to as CitH3 mAb (4 Cit), with the intention to completely block the CitH3 catalyzed by both PAD2 and PAD4.

**Figure 2:**
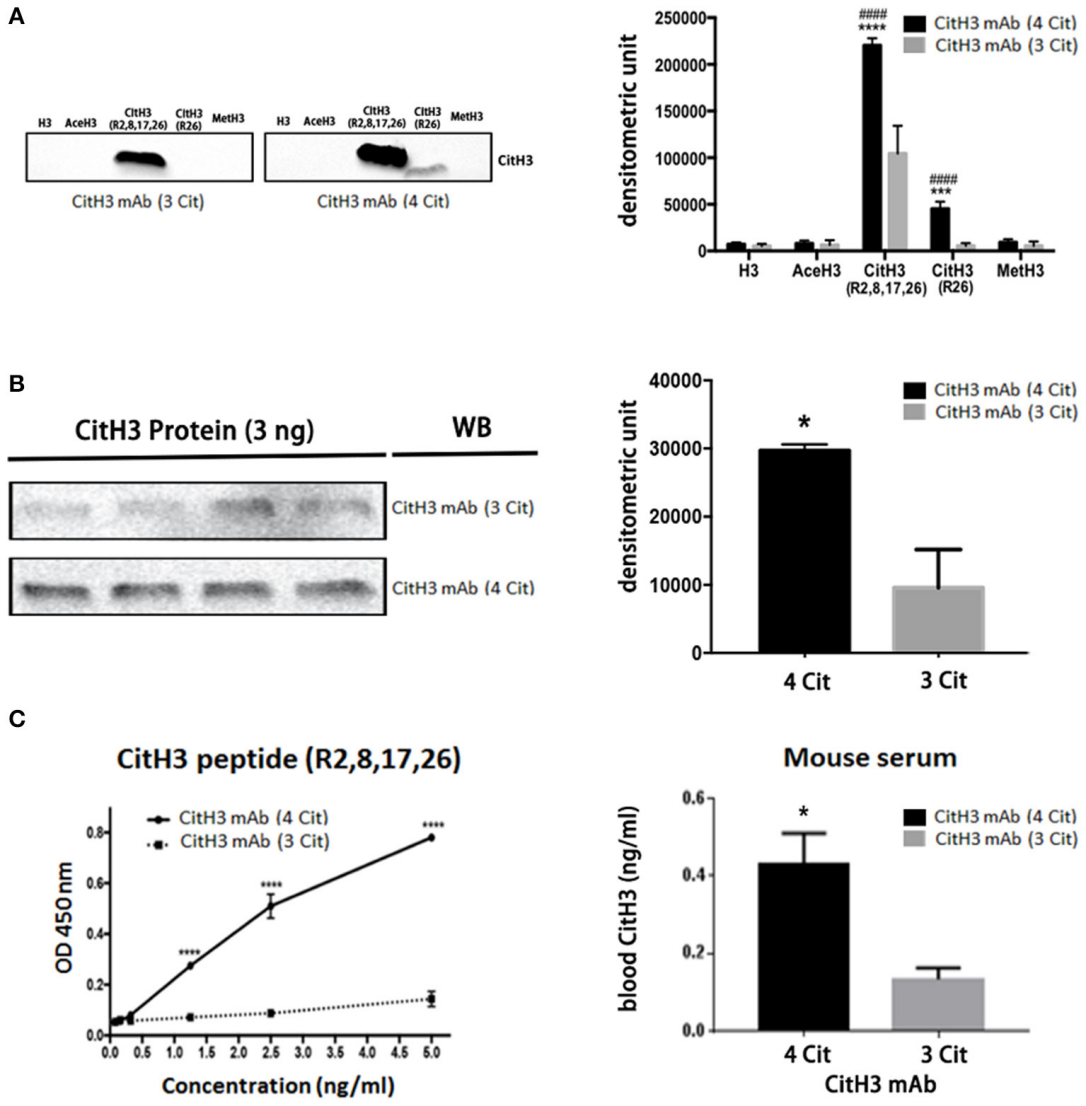
The newly developed anti-CitH3 mAb (4 Cit) recognizes histone H3 citrullinated R2+R8+R17+R26 with higher binding capability than the commercially available anti-CitH3 monoclonal antibody [CitH3 mAb (3 Cit)]. **(A)** Half microgram of five different peptides [H3, AceH3, CitH3 (R2,8,17,26), CitH3 (R26), and MetH3] or **(B)** 3 ng CitH3 protein were submitted to SDS-polyacrylamide gel electrophoresis and transferred onto a nitrocellulose membrane. Then, membranes were probed with the same concentration of CitH3 mAb (4 Cit) or CitH3 mAb (3 Cit) (2 μg/ml). Other immunoblotting conditions were kept the same. Signal intensity was analyzed using Image Studio Lite and presented as mean ± SEM (*n* = 4/group). **(C)** Half microgram of CitH3 mAb (4 Cit) or CitH3 mAb (3 Cit) was coated per well in 96-well plates and incubated for 2 h at room temperature (RT) with CitH3 peptide (left panel), and or serum (1:1 diluted, right panel) from endotoxic mice, followed by anti-CitH3 polyclonal antibody incubation and then donkey anti-rabbit HRP-conjugated IgG. 3,3′, 5,5′-Tetramethylbenzidine was utilized to develop the plate for 30 min at RT in the dark before adding stop solution. Absorbance was measured at 450 nm and presented as mean ± SEM (*n* = 3/group). *p < 0.05 compared to CitH3 mAb (3 Cit); ***p < 0.001 compared to CitH3 mAb (3 Cit); ****p < 0.0001 compared to CitH3 mAb (3 Cit); ^*####*^*p* < 0.0001 compared to H3 incubated with CitH3 mAb (4 Cit). WB, Western blot.

To ensure the quality of the CitH3 mAb (4 Cit), we performed both immunoblotting and ELISA to test its specificity and capability for CitH3 recognition, compared with a commercial CitH3 mAb (3 Cit) (Figure 2). Immunoblotting was performed under the same experimental conditions, including concentration of antibodies, incubation time, and same-time exposure. As shown in Figure 2A, both CitH3 mAb (4 Cit) and CitH3 mAb (3 Cit) had a high specificity for CitH3. However, no band was detected for H3 (non-modified H3), AceH3 (acetylated H3), or MetH3 (methylated H3). For CitH3 detection, CitH3 mAb (3 Cit) only showed staining for CitH3 (R2+R8+R17+R26) peptide; however, no signal was found for CitH3 (R26) peptide. The CitH3 mAb (4 Cit) specifically detected histone H3 citrullinated R26, in accordance with our expectation. Furthermore, using CitH3 mAb (4 Cit), a stronger blot signal appeared for the CitH3 (R2+R8+R17+R26) peptide [densitometry unit, CitH3 mAb (4 Cit) vs. CitH3 mAb (3 Cit): 220,413.7 ± 4,444.8 vs. 104,416.3 ± 17,285.4; *p* < 0.0001]. Consistent with the peptide immunoblotting results, CitH3 mAb (4 Cit) gave a higher signal (three-fold) for the CitH3 protein [Figure 2B, densitometry unit, CitH3 mAb (4 Cit) vs. CitH3 mAb (3 Cit): 29,689 ± 919.8 vs. 9,596 ± 5,598; *p* < 0.05]. Since the experimental conditions of immunoblotting can only be roughly controlled, ELISA was used to further confirm the superiority of CitH3 mAb (4 Cit) in comparison with CitH3 mAb (3 Cit). As shown in Figure 2C, CitH3 mAb (4 Cit) showed significantly higher optical density (OD) than the CitH3 mAb (3 Cit) in detecting CitH3 peptide at various concentrations. Most importantly, a higher concentration of CitH3 was detected using CitH3 mAb (4 Cit) compared to the CitH3 mAb (3 Cit) in a mouse model of endotoxic shock (the right panel of Figure 2C).

Taken together, these findings suggest that CitH3 mAb (4 Cit) not only has specificity comparable to CitH3 mAb (3 Cit) but also possesses higher binding capabilities and recognizes more citrulline residues such as histone H3 citrullinated R2+R8+R17.

### The CitH3 mAb (4 Cit) Improves Survival Compared to CitH3 mAb (3 Cit) Following LPS-Induced Endotoxic Shock

**Figure 3:**
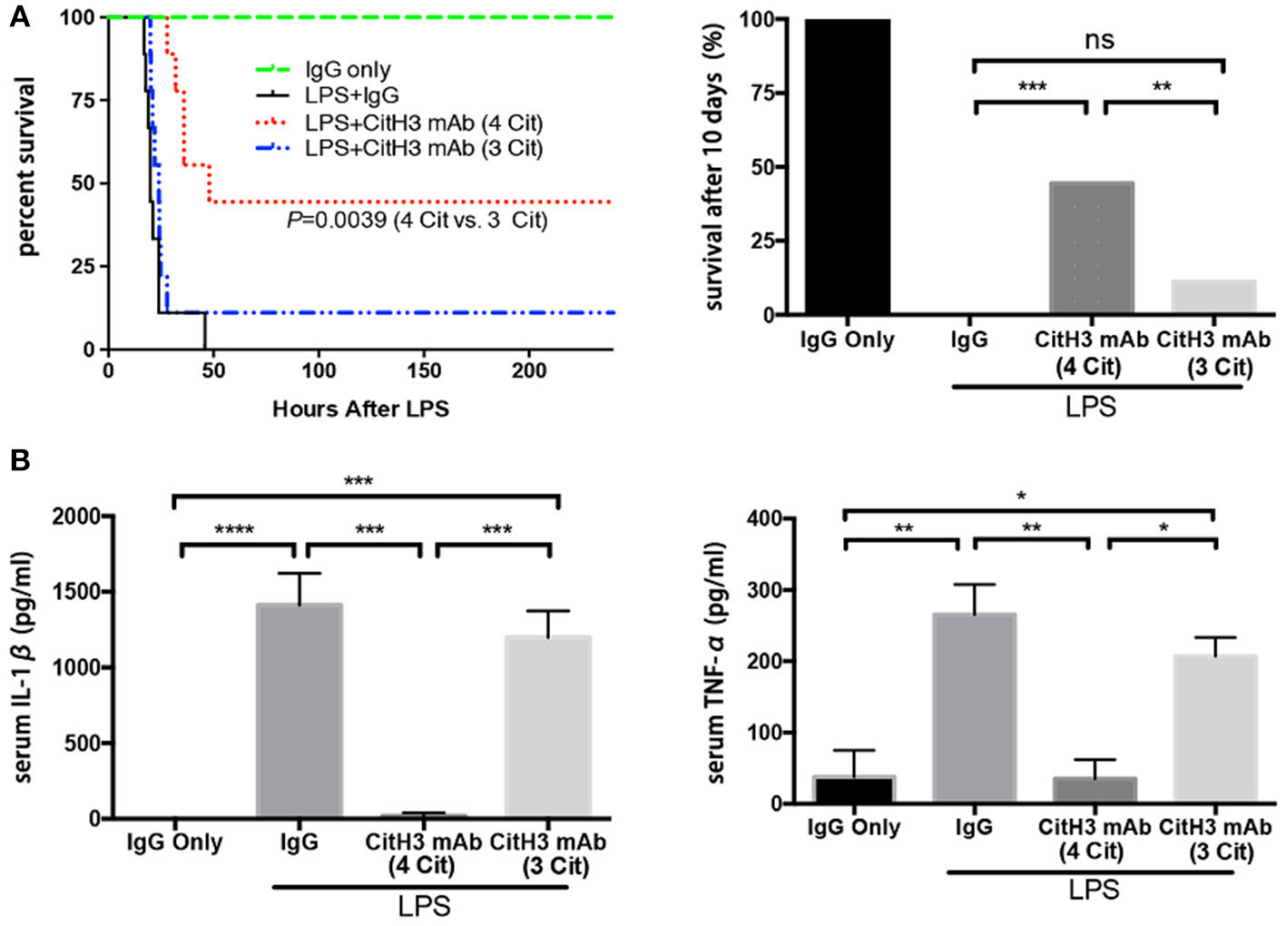
The CitH3 mAb (4 Cit) improves survival and attenuates serum cytokines compared to the CitH3 mAb (3 Cit) in a mouse model of lethal endotoxic shock. C57BL/6J mice were randomized to injection: (1) IgG only (20 mg/kg), (2) LPS (20 mg/kg) + mouse IgG (20 mg/kg), LPS + CitH3 mAb (4 Cit) (~20 mg/kg), LPS + CitH3 mAb (3 Cit) (~20 mg/kg). **(A)** Survival was monitored for 10 days (*n* = 9/group). Kaplan–Meier curves were used for survival rate analysis. The CitH3 mAb (4 Cit) significantly improved mouse survival compared to the LPS + mouse IgG group (*p* = 0.0004) and to the LPS + CitH3 mAb (3 Cit) group (*p* = 0.0039). There was no survival difference between the LPS + IgG group and the LPS + CitH3 mAb (3 Cit) group. **(B)** In another cohort (*n* = 3/group), blood and organs were harvested at 12 h after LPS injection. Serum levels of IL-1β and TNF-α were measured using ELISA. Data are presented as mean ± SEM (*n* = 3/group). *p < 0.05; **p < 0.01; ***p < 0.001; ****p < 0.0001. ns, non significance.

After validation of the CitH3 mAb (4 Cit), we evaluated whether the new antibody could improve survival in a mouse model of LPS-induced endotoxic shock. It has been shown previously that CitH3 appears within 30 min of endotoxic shock (18, 23). In the present study, mice were intravenously administrated anti-CitH3 mAb immediately after LPS injection. The potential non-specific therapeutic effects of immunoglobulin were controlled in a parallel cohort by giving mouse IgG treatment. Survival was monitored for 10 days (Figure 3A). The CitH3 mAb (4 Cit) treatment significantly improved the survival rate of endotoxic mice compared to either LPS + IgG (44.44 vs. 0%; *p* = 0.0004) or LPS + CitH3 mAb (3 Cit) group (44.44 vs. 11.11%; *p* = 0.0039). In addition, a significant decrease was observed in the serum IL-1β and TNF-α levels with CitH3 mAb (4 Cit) treatment at the 12-h time point, compared to either the LPS + IgG group (*p* < 0.001 and *p* < 0.01, respectively) or the LPS + CitH3 mAb (3 Cit) group (*p* < 0.01 and *p* < 0.05, respectively) (Figure 3B).

These data revealed that the new CitH3 mAb (4 Cit) could effectively protect the mice from lethal endotoxic shock, and significantly ameliorate the pro-inflammatory effects caused by LPS administration, in comparison with CitH3 mAb (3 Cit) and mouse IgG.

### The CitH3 mAb (4 Cit) Protects Against LPS-Induced ALI

**Figure 4:**
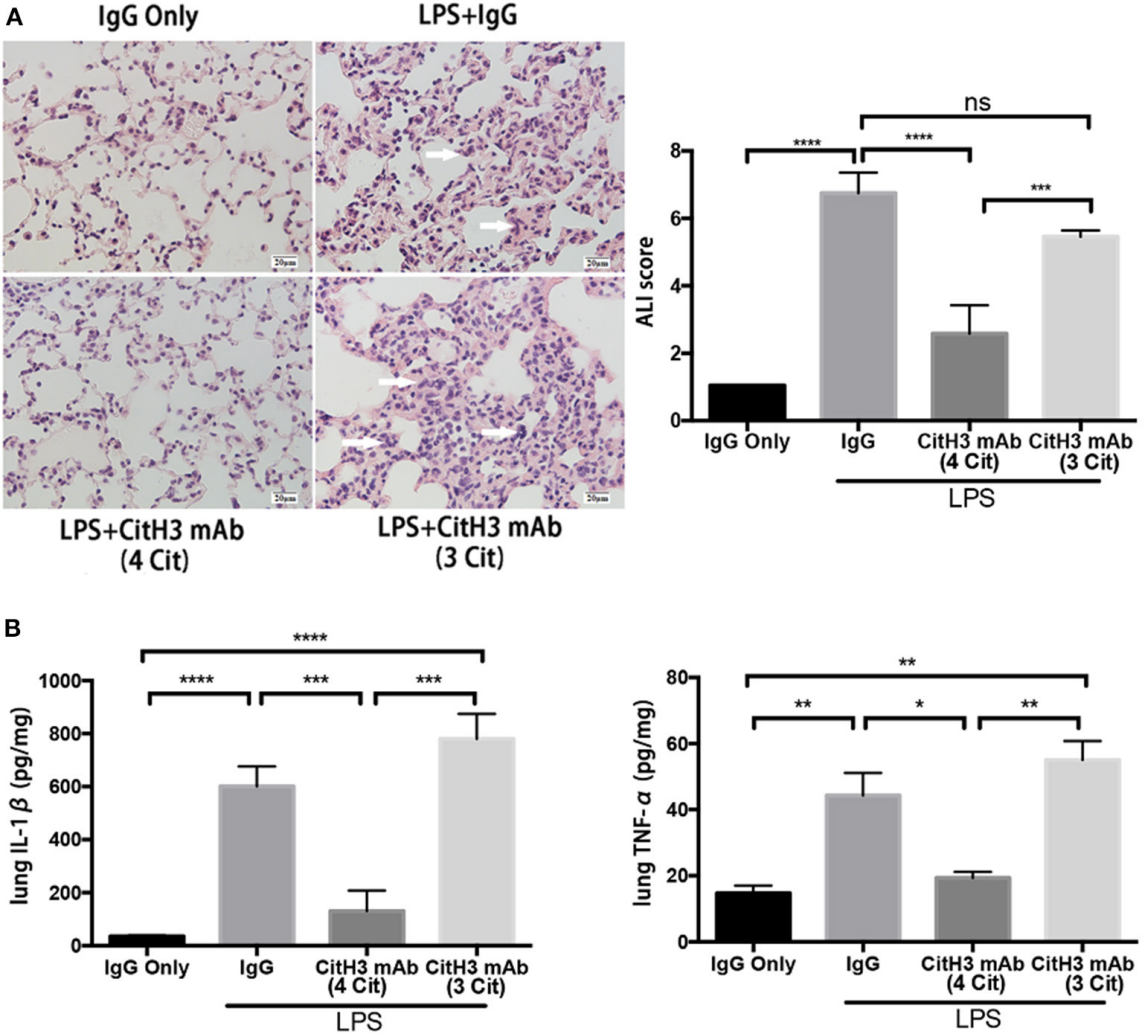
The CitH3 mAb (4 Cit) ameliorates acute lung injury in lethal endotoxic shock. Mice were randomly divided into four groups: (1) IgG only (20 mg/kg), (2) LPS (20 mg/kg) + IgG (20 mg/kg), (3) LPS + CitH3 mAb (4 Cit) (20 mg/kg), and (4) LPS + CitH3 mAb (3 Cit) (20 mg/kg) (*n* = 3/group). Blood and organs were harvested at 12 h after LPS injection. **(A)** Representative hematoxylin and eosin staining of mouse lung sections is shown, and histological analysis of acute lung injury (ALI) was graded by a blinded pathologist and presented as ALI score (mean ± SEM, *n* = 3/group). White arrows indicate inflammatory changes. **(B)** IL-1β and TNF-α of lung homogenates were determined by ELISA. Cytokine levels were normalized by protein concentration and were significantly lower in CitH3 mAb (4 Cit)-treated mice (mean ± SEM, *n* = 3/group). *p < 0.05; **p < 0.01; ***p < 0.001; ****p < 0.0001. ns, non significance.

Sepsis causes end-organ dysfunction, and septic patients are particularly at the risk of developing ALI (31). Moreover, ALI is one of the leading causes of mortality in septic patients (32). Therefore, we examined pathological changes in the lung 12 h after LPS insult, using H&E staining. As shown in the left panel of Figure 4A, IgG injected control animals showed normal histology, while the lungs from the LPS + IgG group displayed obvious inflammatory changes: inflammatory infiltrates, pulmonary congestion, edema, alveolar hemorrhage, and thickening of the alveolar wall. The CitH3 mAb (4 Cit) administration markedly ameliorated the histopathology changes induced by LPS. On the contrary, the CitH3 mAb (3 Cit) treatment was unable to protect the lungs against ALI.

The severity of lung injury was further quantitatively evaluated by a pathologist, blinded to the group allocation of the samples, by calculating the ALI score (Figure 4A). Lung septal mononuclear cell/lymphocyte, septal hemorrhage and congestion, neutrophils, alveolar macrophages, alveolar hemorrhage, and alveolar edema were assessed (see Materials and Methods). The ALI score was significantly increased after LPS insult (LPS + IgG vs. IgG: 6.750 ± 0.351 vs. 1.050 ± 0.000; *p* < 0.0001); however, the score was markedly reduced more than two-fold in the CitH3 mAb (4 Cit) treatment group [LPS + CitH3 mAb (4 Cit) vs. LPS + IgG: 2.583 ± 0.483 vs. 6.750 ± 0.3512; *p* < 0.0001]. The effects of CitH3 mAb (3 Cit) were not as strong as the CitH3 mAb (4 Cit). Even though the mean ALI score value of the LPS + CitH3 mAb (3 Cit) group was slightly lower than the LPS + IgG group, there were no statistical difference between the groups [LPS + CitH3 mAb (3 Cit) vs. LPS + IgG: 5.457 ± 0.107 vs. 6.750 ± 0.3512; *p* < 0.0001].

We also measured the levels of IL-1β and TNF-α in the lung homogenate. A significant decrease in lung IL-1β [LPS + CitH3 mAb (4 Cit) vs. LPS + IgG: 130.7 ± 44.67 vs. 601.0 ± 43.47 pg/mg; *p* < 0.001] and TNF-α [LPS + CitH3 mAb (4 Cit) vs. LPS + IgG: 19.33 ± 1.856 vs. 44.33 ± 6.741 pg/mg; *p* < 0.01] after CitH3 mAb (4 Cit) treatment was observed, in accordance with the serum IL-1β and TNF-α changes. However, the CitH3 mAb (3 Cit) could not effectively decrease IL-1β [LPS + CitH3 mAb (3 Cit) vs. LPS + IgG: 780 ± 54.5 vs. 601.0 ± 43.47 pg/mg; ns] or TNF-α [LPS + CitH3 mAb (3 Cit) vs. LPS + IgG: 55.00 ± 5.774 vs. 44.33 ± 6.741 pg/mg; ns]. In these experiments, the sample size may seem low (*n* = 3/group) but the results reach significant difference (*p* < 0.05) based on our statistical analysis.

Taken together, administration of CitH3 mAb (4 Cit), but not CitH3 mAb (3 Cit), was found to protect against LPS-induced ALI and attenuate the inflammatory cytokines.

### Administration of CitH3 mAb (4 Cit) Decreases Serum Levels of dsDNA

Since CitH3 is generally considered a component of NETs, the binding of the anti-CitH3 antibody and the antigen might affect NETs in the circulation.

dsDNA levels were significantly decreased after the CitH3 mAb (4 Cit) treatment [LPS + IgG vs. LPS + CitH3 mAb (4 Cit): 9.047 ± 0.816 vs. 2.537 ± 0.3767 μg/ml; *p* < 0.001]. However, this was not observed with the CitH3 mAb (3 Cit) [LPS + IgG vs. LPS + CitH3 mAb (3 Cit): 9.047 ± 0.816 vs. 10.72 ± 0.68 μg/ml; ns]. Moreover, the CitH3 mAb (4 Cit) treatment group had less dsDNA compared with the 3 Cit mAb [LPS + CitH3 mAb (4 Cit) vs. LPS + 3 Cit mAb: 2.537 ± 0.3767 vs. 10.72 ± 0.68 μg/ml; *p* < 0.0001].

## Discussion

In the current study, we have demonstrated that exposure to CitH3 can increase endothelial cell leakage and induce the formation of NETs through a positive feedback system. We neutralized the circulating CitH3 with a newly developed CitH3 mAb (4 Cit) that strongly binds to four citrulline sites on CitH3, in comparison with the commercial CitH3 mAb (3 Cit) that binds to only three citrulline sites. We found that injection of CitH3 mAb (4 Cit) markedly improves survival following LPS-induced lethal endotoxic shock and attenuates pro-inflammatory responses, ALI, as well as NET formation. Our antibody fills the gap in the field of citrullinated histone that was reported by Neeli and Radic, “current challenges and limitations in antibody-based detection of citrullinated histones,” in *Frontiers in Immunology* (28). In addition, we believe that CitH3 can be a potential target for directed therapies in endotoxic patients in the future.

Neutrophils are the most abundant innate immune cells in humans, and their functions include phagocytosis of pathogens, release of multiple inflammatory mediators, and induction of NET formation. Release of NETs by neutrophils was first discovered by Brinkman et al. (4), and has attracted significant interest in recent years. NETs have been found to play key roles in various diseases including sepsis, tumors, systemic lupus erythematosus (SLE), rheumatoid arthritis (RA), gout, and others (15). NETs also play a key role in sepsis. NETs are able to trap microbes and keep them in a restricted area with high concentrations of anti-microbial agents, which are released as components of NETs (33). DNA itself has been reported to possess anti-microbial activities (34). Histones and histone-like proteins also function as microbial-combating reagents in a variety of species (35). However, unless carefully controlled, excessive NETs and components of NETs can be detrimental in sepsis. Histones and citrullinated histones are both components of NETs and have been found to be elevated in sepsis. Histones bind to endothelial cells, cause an increase in endothelial cell permeability and Ca^2+^ influx, eventually leading to cell death (36). Histones also promote thrombosis through impairing thrombomodulin-dependent protein C activation (37, 38). In addition, extracellular histones have been found to mediate liver injury through toll-like receptor 2 (TLR2) and TLR4 (39). Unlike histones, the role of citrullinated histones in diseases remains largely unknown. CitH3 release has been detected in blood and peritoneal fluid in sepsis (17, 18, 23); however, the effects of CitH3 production and its pathophysiologic roles in sepsis are unclear.

**Figure 5:**
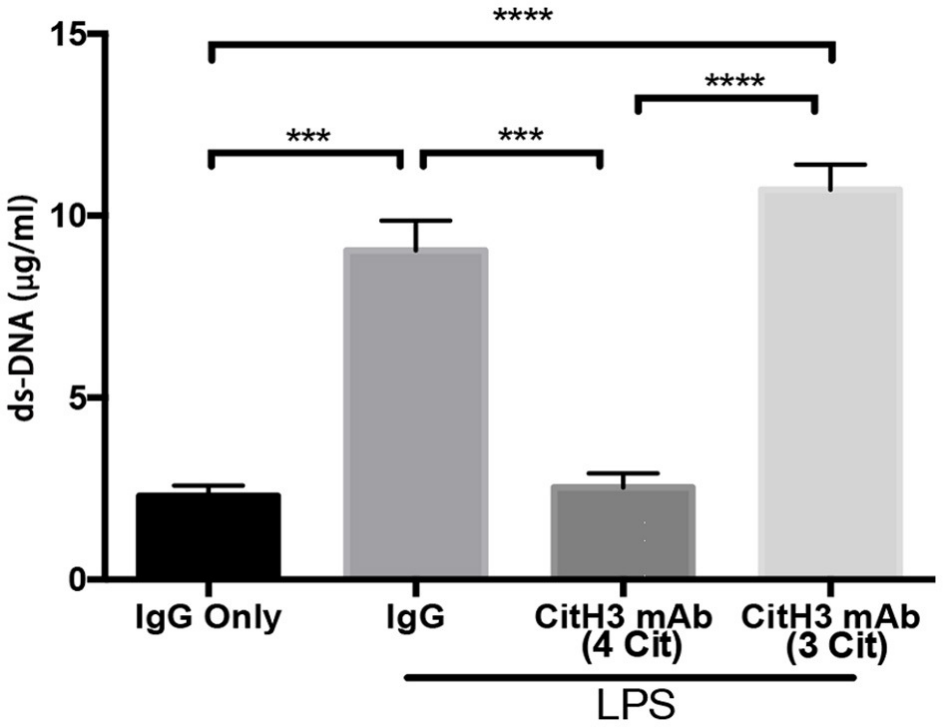
The CitH3 mAb (4 Cit) decreases serum dsDNA. Mice were randomly divided into four groups: (1) IgG only (20 mg/kg), (2) LPS (20 mg/kg) + IgG (20 mg/kg), (3) LPS + 4 CitH3 mAb (20 mg/kg), and (4) LPS + CitH3 mAb (3 Cit) (20 mg/kg) (*n* = 3/group). Blood was harvested at 12 h after LPS injection. dsDNA was measured using PicoGreen assay kit and expressed as mean ± SEM (*n* = 3/group). ***p < 0.001; ****p < 0.0001.

There are multiple ways to induce septic response in animals: (1) LPS, bacterial, or cecal slurry injection; (2) endogenic protection barrier model, such as CLP and colon ascendens stent peritonitis (CASP); and (3) extra-abdominal models of sepsis: pneumonia and urosepsis (40). Due to its stability, repeatability, and long track record (41), we selected LPS-induced endotoxic shock as a starting model to test the therapeutic efficacy of the CitH3 mAb (4 Cit). The commercial anti-CitH3 mAb (3 Cit) detected H3 citrullinated R2+R8+R16 and served as a control. Improved survival was observed in the CitH3 mAb (4 Cit)-treated mice compared to the CitH3 mAb (3 Cit) or IgG-treated endotoxic mice (Figure 3A). Several reasons might contribute to the better outcome after CitH3 mAb (4 Cit) administration: (1) less endothelial leakage (Figure 1A); (2) inhibited inflammatory responses (Figure 3B, Figure 4B); (3) attenuated ALI (Figure 4A); and (4) decreased formation of NETs (Figure 5).

Endothelial dysfunction is the main reason for multiple organ failure in sepsis, since it causes tissue edema, disarrangement of hemostasis, and vasomotor control, eventually leading to death (42). In this study, the CitH3 peptide was shown to increase permeability of HUVECs *in vitro* (Figure 1A). In addition, infusion of CitH3 protein has been shown to induce an extravasation of fluorescent-labeled albumin across mouse mesenteric micro vessels without cell death (25). These observations suggest that CitH3 might be one of the causes for vascular leakage and organ edema in sepsis.

Release of cytokines, for example, IL-1β and TNF-α, can activate and recruit effector inflammatory cells to the site of infection. However, excessive or deregulated cytokine profile is a common occurrence in sepsis, exerting harmful effects both systemically and locally. The level of TNF-α in septic patients correlates with fatal outcome (43). Persistent elevation of cytokine concentrations in patient plasma, including TNF-α and IL-1β, is associated with poor prognosis in septic patients with acute respiratory distress syndrome (ARDS) (44). The fact that CitH3 mAb (4 Cit) can decrease the levels of circulating IL-1β and TNF-α (Figure 3B) could attenuate the adverse effects that result from excessive cytokine release.

ALI is one of the most frequent complications to develop in septic patients and among the leading causes of deaths in these patients (32). In our experimental LPS model, survival correlated strongly with reduced lung injury. Endotoxic mice treated with the CitH3 mAb (4 Cit) had better lung histology (lower ALI score) as well as much less pro-inflammatory cytokines in lung, compared to the mice treated with IgG or the CitH3 mAb (3 Cit) (Figure 4A). The alleviated ALI might be one of the mechanisms responsible for the significantly better survival in the CitH3 mAb (4 Cit)-treated mice. Endotoxin/sepsis-induced inflammatory responses are important defense mechanisms in disease conditions; however, they can also contribute to the development of ALI. Endothelial cells, epithelial cells, resident alveolar macrophages, and neutrophils secrete cytokines and chemokines, leading to an increase in tissue inflammation and subsequent cellular damage. Among the cytokines, IL-1β and TNF-α are major cytokines released into the alveolar spaces of patients with ARDS (45). In our study, we also found elevated levels of IL-1β and TNF-α in lung homogenates (Figure 4B). IL-1β and TNF-α are known to exaggerate the inflammatory responses initiated by endotoxin, which could result in adverse consequences. We have shown that administration of CitH3 mAb (4 Cit) significantly attenuated the local concentrations of IL-1β and TNF-α in the lung, which could be protective against development of ALI.

Our results (Figure 1C) also suggest that CitH3 can induce NET formation through a positive feedback mechanism. There is plenty of evidence showing the detrimental role of NETs (15). NETs can damage epithelium, endothelium, and various tissues including liver and lung (36, 46–48); moreover, NETs promote thrombosis, leading to vasculature occlusion (49–52). The level of circulating dsDNA has been shown to correlate with disseminated intravascular coagulation (DIC) score and predicts DIC independently (53). In this study, the CitH3 mAb (4 Cit) efficiently inhibited this positive feedback loop, leading to less formation of CitH3/NETs (Figure 5). The underlying mechanism may be that the mAb (4 Cit) binds to CitH3 and forms immune complex, which are further cleared by immune system (54, 55). The DNA backbone and associated proteins may also be eliminated along with the ICs. Therefore, neutralizing CitH3 directly decreased free CitH3 in circulation, as well as blocking the positive feedback to inhibit CitH3 self-amplification, thus preventing the endothelial dysfunction caused by CitH3.

The major difference between the CitH3 mAb (4 Cit) and the CitH3 mAb (3 Cit) is thought to be secondary to their relative abilities to neutralize the CitH3 protein. The newly developed CitH3 mAb (4 Cit) recognizes four citrulline sites: H3 citrullinated R2+R8+R17+R26, while the CitH3 mAb (3 Cit) is only designed for three citrulline spots: H3 citrullinated R2+R8+R17. As demonstrated by immunoblotting and ELISA (Figure 2), the CitH3 mAb (4 Cit) specifically recognized H3 R26 citrullination and had a stronger capability to bind CitH3. In addition, different citrullination may correspond to different PADs. Among the five PAD isoforms that have been discovered, only PAD2 and PAD4 have been demonstrated by several lines of evidence to translocate from cytosol to nucleus and citrullinate histone H3. We reasoned that CitH3 in sepsis originated from PAD2 or PAD4 pathways, or both. It has been reported that H3 R26 is a valid target for PAD2, but not PAD4 (30). Therefore, the CitH3 mAb (4 Cit) is suspected to bind and neutralize more circulating CitH3. The one citrulline difference partly contributes to the binding ability discrepancy between these two antibodies.

Wang et al. have demonstrated in *Science* that PAD4 deiminates three arginine residues on H3 at Arg 2, Arg 8, and Arg 17 *in vitro*, and two arginine residues on H3 at Arg 8 and Arg 17 *in vivo* (29). It is not clear what causes the different citrullinations. Basically, an enzyme's active site binds substrates and plays a role in catalysis. It is conceivable that some proteins might bind to histone H3 in the *in vivo* condition and therefore prevent H3 from citrullination by PAD4.

Our interpretations regarding differential PAD2/PAD4 involvement in CitH3 production is supposed not only by the data from this study but also by previous reports by other investigators (29). These findings will need additional verification in the future, and more mechanistic experiments will have to be performed to fully understand this complex process.

In this study, we decided to use the histone H3 peptide for several reasons. *First*, the N-terminal tail (N-tail) of histone H3, which protrudes beyond the nucleosome DNA (56), is regulated by multiple posttranslational modifications (PTMs) (57). Focusing on citrullination of the N-tail, we synthesized N-terminal CitH3 peptide, instead of CitH3 protein, to ensure that histone H3 is citrullinated at the N-terminus of four arginine residues. *Second*, the commercial CitH3 (citrulline R2+R8+R17) (58, 59) antibodies, including Item 9003062 (Cayman Chemical, Ann Arbor, MI) and ab5103 (abcam, Cambridge, MA), are generated with CitH3 N-tail peptide. To compare our CitH3 mAb (4 Cit) to the commercial CitH3 mAb (3 Cit), it is more appropriate to use the N-terminal CitH3 peptide as an antigen. *Third*, histone H3 (citrullinated or non-citrullinated) epitope(s) can be cleaved off at the N-terminus of H3 (60). To determine the effect of the N-tail of H3 and CitH3 *in vitro*, it is logical to use the N-terminal H3/CitH3 peptide as the stimulus.

There are several limitations to this study. We only used a murine endotoxic shock model to test the therapeutic effects of the CitH3 mAb (4 Cit); however, further evaluation is required in different models of bacterial and polymicrobial infections. In addition, more mechanism studies are needed. For example, further exploration of CitH3 in diverse cell signaling pathways is helpful to better explain how the CitH3 mAb (4 Cit) improves outcomes in endotoxic shock.

In conclusion, we have demonstrated that CitH3 can increase endothelial cell leakage and self-amplify through positive feedback. Neutralizing circulating CitH3 with the CitH3 mAb (4 Cit) markedly increases mouse survival from LPS-induced lethal endotoxic shock, likely secondary to the CitH3 mAb (4 Cit) binding to CitH3 and specifically recognizing H3 R26 citrullination. The CitH3 mAb (4 Cit) also attenuates pro-inflammatory responses, ALI, as well as NET formation, compared to the CitH3 mAb (3 Cit). Overall, these results suggest that sufficient neutralization of CitH3 might be a promising therapeutic strategy for endotoxic shock.

## Data Availability Statement

All datasets generated for this study are included in the article.

## Ethics Statement

The protocol for the animal experiments was approved by the University of Michigan Institutional Animal Care and Use Committee (PRO00008861). All experiments complied with animal welfare and research regulations.

## Author Contributions

YLi and HA designed this study. QD, BP, and YLia performed the experiments and collected and analyzed data. BL, YT, AW, and JZ provided experimental support. XD pathologically examined the lung tissues. QD wrote the manuscript, which was critically reviewed and revised by YLi, HA, NM-V, ZW, and AW. All authors read and approved the final manuscript.

### Conflict of Interest

YLi and HA are inventors on a patent application related to the CitH3 mAb (4Cit). The remaining authors declare that the research was conducted in the absence of any commercial or financial relationships that could be construed as a potential conflict of interest.

## Abbreviations

CitH3 mAb (4 Cit): anti-histone H3 (citrullinated R2+R8+R17+R26) monoclonal antibody
CitH3 mAb (3 Cit): anti-histone H3 (citrullinated R2+R8+R17) monoclonal antibody.
